# Effects of angiotensin II combined with asparaginase and dexamethasone on the femoral head in mice: A model of steroid-induced femoral head osteonecrosis

**DOI:** 10.3389/fcell.2022.975879

**Published:** 2022-09-08

**Authors:** Jiahe Liu, Chenzhi Li, Fan Yang, Minde Li, Baolin Wu, Haojie Chen, Shaopeng Li, Xiuzhi Zhang, Jiahui Yang, Yan Xia, Mingjian Wu, Yancheng Li, Baoyi Liu, Dewei Zhao

**Affiliations:** ^1^ Department of Orthopedics, Affiliated Zhongshan Hospital of Dalian University, Dalian, Liaoning, China; ^2^ Institute of Metal Research Chinese Academy of Sciences, Shenyang, Liaoning, China

**Keywords:** animal model, femoral head osteonecrosis, angiotensin II, asparaginase, dexamethasone

## Abstract

**Background:** To study the pathogenesis of steroid-induced femoral head osteonecrosis, an ideal animal model is very important. As experimental animals, mice are beneficial for studying the pathogenesis of disease. However, there are currently few mouse models of steroid-induced femoral head osteonecrosis, and there are many questions that require further exploration and research.

**Purposes:** The purpose of this study was to establish a new model of osteonecrosis in mice using angiotensin II (Ang II) combined with asparaginase (ASP) and dexamethasone (DEX) and to study the effects of this drug combination on femoral head osteonecrosis in mice.

**Methods:** Male BALB/c mice (*n* = 60) were randomly divided into three groups. Group A (normal control, NC) was treated with physiological saline and given a normal diet. Group B (DEX + ASP, DA) was given free access to food and water (containing 2 mg/L DEX) and subjected to intraperitoneal injection of ASP (1200 IU/kg twice/week for 8 weeks). Group C (DEX + ASP + Ang II, DAA) was treated the same as group B, it was also given free access to food and water (containing 2 mg/L DEX) and subjected to intraperitoneal injection of ASP (1200 IU/kg twice/week for 8 weeks), but in the 4th and 8th weeks, subcutaneous implantation of a capsule osmotic pump (0.28 mg/kg/day Ang II) was performed. The mice were sacrificed in the 4th and 8th weeks, and the model success rate, mouse mortality rate, body weight, blood lipids, coagulation factors, histopathology, and number of local vessels in the femoral head were evaluated.

**Results:** DAA increased the model success rate [4th week, 30% (DA) vs. 40% (DAA) vs. 0% (NC); 8th week, 40% (DA) vs. 70% (DAA) vs. 0% (NC)]. There was no significant difference in mortality rate between the groups [4th week, 0% (DA) vs. 0% (DAA) vs. 0% (NC); 8th week, 5% (DA) vs. 10% (DAA) vs. 0% (NC)]. DAA affected mouse body weight and significantly affected blood lipids and blood coagulation factors. DAA reduces the number of blood vessels in the femoral head and destroys the local blood supply.

**Conclusion:** Angiotensin II combined with asparaginase and dexamethasone can obviously promote the necrosis of femoral head and provide a new idea for the model and treatment of osteonecrosis.

## Introduction

There are many causes of nontraumatic avascular osteonecrosis, including high blood pressure, thrombosis, alcohol consumption, chemotherapy, and excessive glucocorticoid (GC) treatment (Jones, 2000; Assouline-Dayan et al., 2002; Laroche, 2002; Chao et al., 2003; Suzuki et al., 2008). The use of GCs is an important factor that induces nontraumatic avascular osteonecrosis, but the underlying mechanisms are still unclear (Kerachian et al., 2009). To further study steroid-induced femoral head osteonecrosis, animal models are needed. However, no ideal animal model of steroid-induced femoral osteonecrosis exists; therefore, it is necessary to improve the current models.

The ability to reduce the number of blood vessels and disrupt the local blood supply is considered an important factor in GC-induced femoral head osteonecrosis (Kerachian et al., 2009; Weinstein, 2012). Recent studies have shown that GCs can directly harm vascular endothelial cells, disrupt the coagulation-fibrinolys is system and ultimately form thromboses in the femoral head, severely reducing the blood supply to the trabecular bone (Kerachian et al., 2006; Kerachian et al., 2009; Weinstein, 2012). GCs can directly reduce the production of blood vessels in bone *in vivo*. Micro-CT scanning shows that GCs can directly reduce the number of blood vessels in and the surface area of the spine and femur in mice (Weinstein et al., 2010). Blood vessels provide nutrition to bones and are important in bone remodeling (Wan et al., 2010). To enhance the GC-induced osteonecrosis rate, it is important to destroy blood vessels and inhibit angiogenesis. Asparaginase (ASP) is commonly used in the treatment of leukemia and can inhibit angiogenesis, influence the local blood supply and affect vascular endothelial cell damage. Increasing evidence shows that ASP increases the risk of femoral head osteonecrosis (Yang et al., 2008; Batool et al., 2016). Liu C et al. established a mouse femoral head osteonecrosis model using ASP and dexamethasone (DEX), with a success rate of 43%. Compared with that of DEX alone, the femoral head osteonecrosis rate increased significantly in this model (Liu et al., 2016). Angiotensin II (Ang II), which is converted from Ang I, is a common clinical drug that raises blood pressure. Ang II generally causes deleterious effects, such as vasoconstriction, endothelial dysfunction, inflammation, fibrosis, thrombosis, and angiogenesis (Touyz and Montezano, 2018). In view of the impact of ASP and Ang on local blood transport in bone tissue and damage to the vascular endothelium, endothelial cells can directly influence the formation of new blood vessels (Kusumbe et al., 2014). Referring to previous studies (Liu et al., 2016), adjusting by pre-experiment, we finally determined the experimental dosage of ASP and DEX. We choose 0.28 mg/(kg•d) Ang II as the experimental dose because previous studies showed that 0.28 mg/(kg•d) Ang II could not cause hypertension in mice, but could increase the necrosis rate of femoral head in mice (Dinh et al., 2017). The purpose of this study was to confirm whether DAA could increase the rate of femoral head osteonecrosis in mice compared with that in mice treated with ASP combined with DEX. Analyses of mouse blood lipids and blood coagulation factors, hematoxylin and eosin (H&E) staining of the femoral head, and systemic perfusion angiography were used in this study.

## Materials and methods

Male BALB/c mice (*n* = 60) were randomly divided into three groups: group A (normal group), group B [DEX (2 mg/L) +ASP (1200 IU/kg), DA]; and group C (DEX + ASP + Ang II, DAA). In the 4th and 8th weeks, 10 mice were sacrificed in each group. Serum samples from the mice were used to detect blood lipids and coagulation factors, and the local blood vessel volume of the femoral head was detected by perfusion angiography of the femoral head. The femoral heads of the mice were used to obtain pathological tissue sections to determine the success rate of the model.

### Breeding, management, and grouping of mice

Male BALB/c mice (body weight, 20.5–25.4 g; age, 3 months; *n* = 60) were provided by Dalian Medical University Laboratory Animal Center (Dalian, China). The mice were kept in an environment with controlled temperature and humidity (temperature, 22.1°C; humidity, 40%–60%; atmospheric CO_2_), with free access to standard food and water and a light/dark cycle of 12 h. The groups were treated as follows. Group A (normal group) was administered physiological saline and a normal diet. Group B (DEX + ASP, DA) was given free access to food and water. Antibiotics, including tetracycline (Solarbio, 1 g/L), sulfamethoxazole (Northeast Pharmaceuticals Group, Shenyang First Pharmaceutical Co., Ltd., 600 mg/L) and trimethoprim (Northeast Pharmaceuticals Group, Shenyang First Pharmaceutical Co., Ltd., 120 mg/L) were used to prevent infections caused by hormones and were administered in a water bottle once every 3.5 days; these drugs had no effect on osteonecrosis (He et al., 2016). The mice in group B were also given intraperitoneal injections of ASP (1200 IU/kg) twice/week for 8 weeks (Hofbauer et al., 2001). Group C (DEX + ASP + Ang II, DAA) was given free access to food and water (the same food and water as the DA group) and intraperitoneal injections of ASP (1200 IU/kg) twice/week for 8 weeks. In addition, at the beginning of the experiment, subcutaneous implantation of a capsule osmotic pump (ALZET, model 2004, United States), which was set to pump Ang II at 0.28 mg/kg/day for 8 weeks, was performed (Dinh et al., 2017).

This study was approved by the animal management committee of the local government of Dalian, China. All experimental procedures were performed in accordance with the guidelines formulated by the Laboratory Animal Center of Dalian University (Dalian).

### Pump implantation

Once the animal was anesthetized, the skin over the mid-scapular region was shaved and washed. A suitable incision was made adjacent to the site chosen for implantation. A hemostat was inserted into the incision, and by opening and closing the jaws of the hemostat, the subcutaneous tissue was spread to create a pocket for the pump [an osmotic mini pump ALZET model 2004, ALZET, United States; slow-pressor dose of Ang II (0.28 mg/kg/d, ab120183, Abcam) for 8 weeks]. The wound was closed with wound clips or sutures. Two clips were typically sufficient.

### Weight measurement and analysis

Before each injection, we measured the weight of the mouse. We determined the weight of each group of mice (as the mean value plus or minus the standard deviation). The health condition of the mice in each group was investigated during the experiment.

### Serum extraction and analysis

Analysis of serum lipids and coagulation factors in mice: In the 4th and 8th weeks of the experiment, 10 mice from each group were anesthetized and sacrificed. Before the mice were sacrificed, blood was collected from the hearts (after anesthesia, the chest wall of the mouse was cut open to expose the heart, and a 1 ml syringe was used to puncture the left apex of the heart and enter the left ventricular cavity to remove approximately 1 ml of fresh blood). The obtained blood was then injected into a 1.5 ml tube (Solarbio) with heparin and centrifuged at 2500 RPM for 15 min. The plasma was extracted, and the indicators were detected by ELISA. For standards, the standard wells were set, and 50 µl of standard solutions with different concentrations were added to the standard wells. Blank control wells did not contain samples or enzyme-labeled reagent, but the other steps were the same. For sample wells, 40 µl of diluted sample was added to the well to be tested on the substrate plate, and then 10 µl was added to the well (the final dilution of the sample was five times). The sample was added to the bottom of the well plate, trying not to touch the wall of the well, and the mixture was gently shaken. Then, 100 µl of enzyme-labeled reagent was added to each well except the blank wells. After sealing the plate with sealing film, the plate was placed at 37°C and incubated for 60 min. Then, 20× concentrated washing liquid was diluted with distilled water for use. The sealing plate membrane was removed carefully, the liquid was discarded, and the plate was shaken to dry. To wash, each well was filled with washing liquid and let stand for 30 s; the liquid was then discarded. This process was repeated five times, and the plate was patted dry. To develop color, 50 µl of color developer A was added to each well, and then 50 µl of color developer B was added. The plate was gently shaken to mix and placed in the dark for 15 min. Then, 50 µl of termination fluid was added to each well to terminate the reaction (the blue color changed to yellow at this point). The absorbance (OD value) of each well was measured by adjusting the blank well to zero and measuring the absorbance at a wavelength of 450 nm for each well in sequence. The determination was made within 15 min after the addition of the termination solution.

### Angiographic analysis of femoral head perfusion

Systemic vascular perfusion imaging and micro-CT scanning of mice: In the 4th and 8th weeks of the experiment, 10 mice in each group were anesthetized, and their hearts were exposed and connected to an infusion pump (China Baoding, Reeve Fluid Technology Co., Ltd., DT15). Heparinized physiological saline, 4% paraformaldehyde, and Microfil (MV-112, Flow Tech, Inc., Carver, MA) were administered into the heart (the pump input speed was 4 ml/min and lasted for 4 min). Next, the mice were stored at 4°C overnight, and the bilateral femoral heads were then extracted, fixed in 10% formalin for 24 h, and decalcified with 10% EDTA for 48–72 h (with a fluid change once every 24 h). Micro-CT scanning was performed at a voxel of 9 microns, 2-D images were rendered with CT Vol, and reconstructed 3-D images of the vasculature were obtained. Then, the femoral head region of the mice was set as “AREA”, and the blood vessels in the femoral head were set as “VOI” (value of interest). The CT value was adjusted to obtain the area of interest and measure the value of each “AREA” and “VOI”. Finally, the results were computed as VOI/AREA (Zhang et al., 2016).

### Femoral head extraction, treatment and H&E staining analysis

Bilateral femurs were obtained from the mice and immediately fixed with 10% formalin for 48 h at room temperature. The samples were decalcified in 14% ethylenediaminetetraacetic acid for 2 weeks and then dehydrated with a gradient of alcohol concentrations (70%, 80%, and 90%). The decalcified samples were embedded in paraffin, and 3-µm-thick sections were prepared for histological analysis through H&E staining. The sections were stained with CAT hematoxylin (No. CATHE-MM; Biocare Medical, LLC, Pacheco, CA, United States) for 1 min and counterstained in alcoholiceosin (No. 17372-87-1; Sigma-Aldrich; Merck KGaA) for 1 min. All procedures were performed at room temperature. The successful induction of femoral head osteonecrosis was defined as a diffuse presence of empty lacunae and trabecular fractures in the femoral head region of the mice or pyknotic nuclei of bone cells in the trabecular bone, accompanied by osteonecrosis of the surrounding bone marrow cells (Yamamoto et al., 1995; Yamamoto et al., 1997; Xu et al., 2017). Histopathological changes associated with femoral head osteonecrosis were examined under a light microscope (Axio Scope A1; Carl Zeiss AG, Oberkochen, Germany) in a blinded manner by three independent investigators.

### Statistical analysis

All the data are presented as the mean ± standard deviation. After verifying a normal distribution and an equal variance, comparisons between groups were performed by ANOVA. Statistical analyses were performed using SPSS software (version 19.0; SPSS Inc., Chicago, IL, United States). *p* < 0.05 was considered to indicate a significant difference.

## Results

### Effect of DAA on weight in mice

**FIGURE 1:**
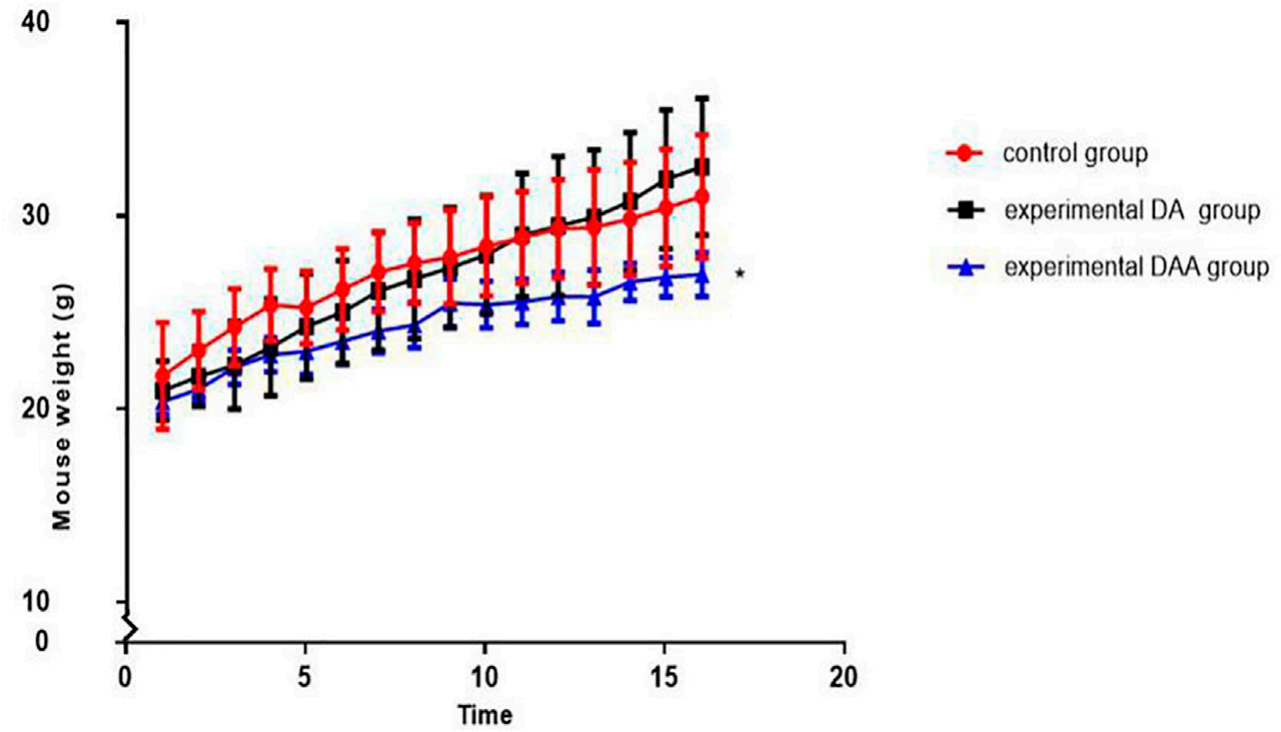
Effects on weight in mice. The mice were weighed twice a week (1/2 weeks interval) for 8 weeks. The mean weight of the mice in the DAA group was significantly lower than that of the mice in the other two groups. However, the mice in each group did not lose weight significantly, reflecting their good health condition. * indicates *p* < 0.05.

DAA significantly inhibited weight gain in mice, and the average weight of the mice in the DAA group was significantly smaller than that of the mice in the other groups. Changes in body weight can reveal the health status of mice and reflect the safety of the model. We weighed the mice before each injection (as shown in Figure 1). By analyzing the weight of the mice in each group, we showed that the mice in the DAA group had the slowest weight gain among the tested groups. However, their weight still increased over time, which indicated that the mice in each group were healthy.

### Effects of DAA on serum lipids and coagulation factors in mice

**FIGURE 2:**
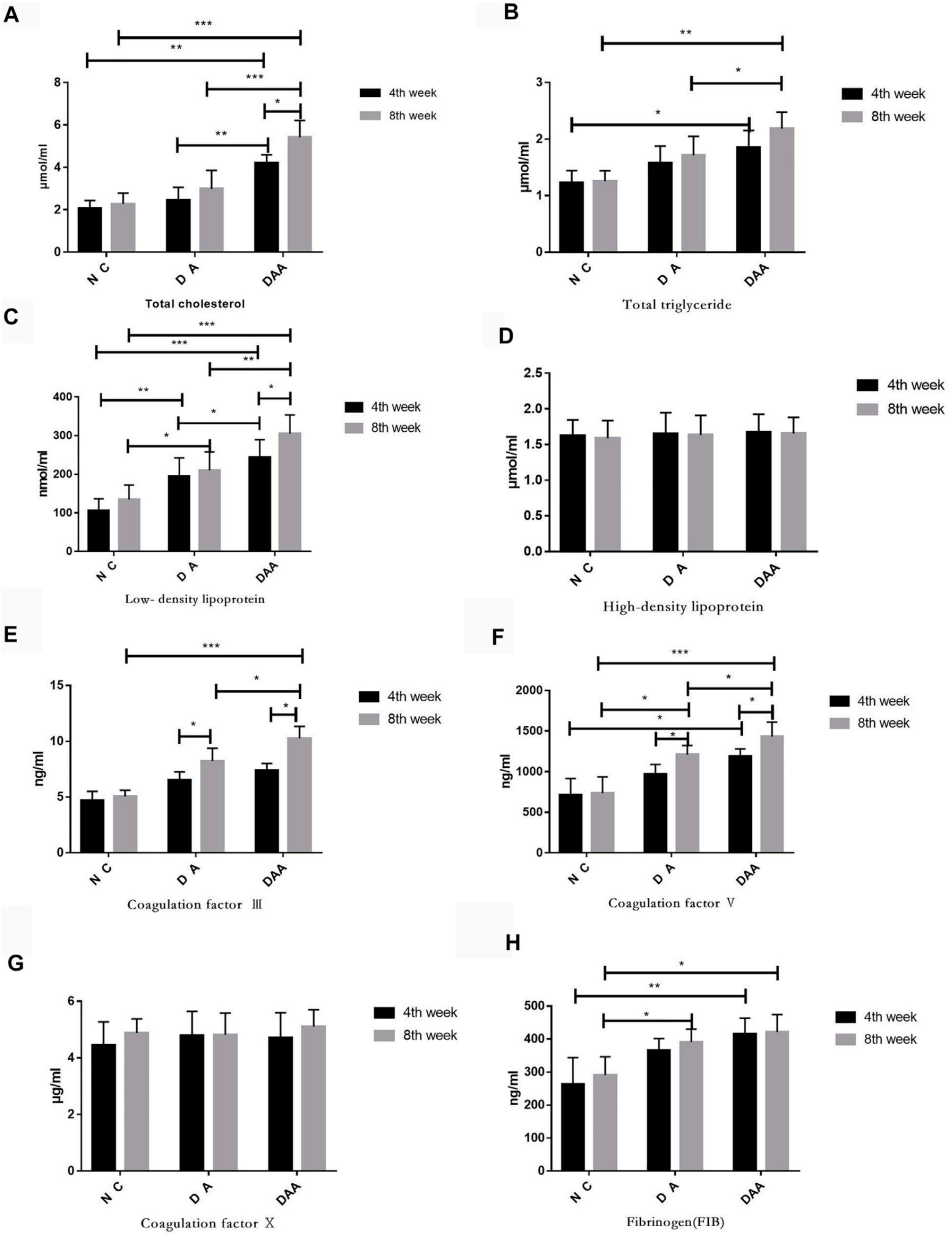
Effects of Ang II combined with ASP on lipids and clotting factors in mice with steroid-induced femoral head osteonecrosis. **(A)** Total cholesterol. **(B)** Total glycerol triglycerides. **(C)** Low-density lipoprotein. **(D)** High-density lipoprotein. There was no significant difference between the groups. **(E)** Clotting factor III. **(F)** Clotting factor V. **(G)** Clotting factor VI. **(H)** Fibrinogen. There was no significant difference between the groups.* indicates *p* < 0.05; ** indicates *p* < 0.01; and *** indicates *p* < 0.001.

DAA significantly affected mouse blood lipid levels and blood coagulation factors and promoted femoral head osteonecrosis. As shown in Figure 2, the data for total cholesterol, total triglycerides, and low-density lipoprotein at the 4th and 8th weeks showed that the DAA group had significantly higher levels than the other two groups (*p* < 0.05). However, high-density lipoprotein exhibited no obvious pattern in any of the groups. Compared with those in the other two groups, the levels of coagulation factors III and V and X in the DAA group were significantly increased (*p* < 0.05), while fibrinogen (FIB) showed no significant differences among the groups.

### Effect of DAA on the number of blood vessels in the femoral head

**FIGURE 3:**
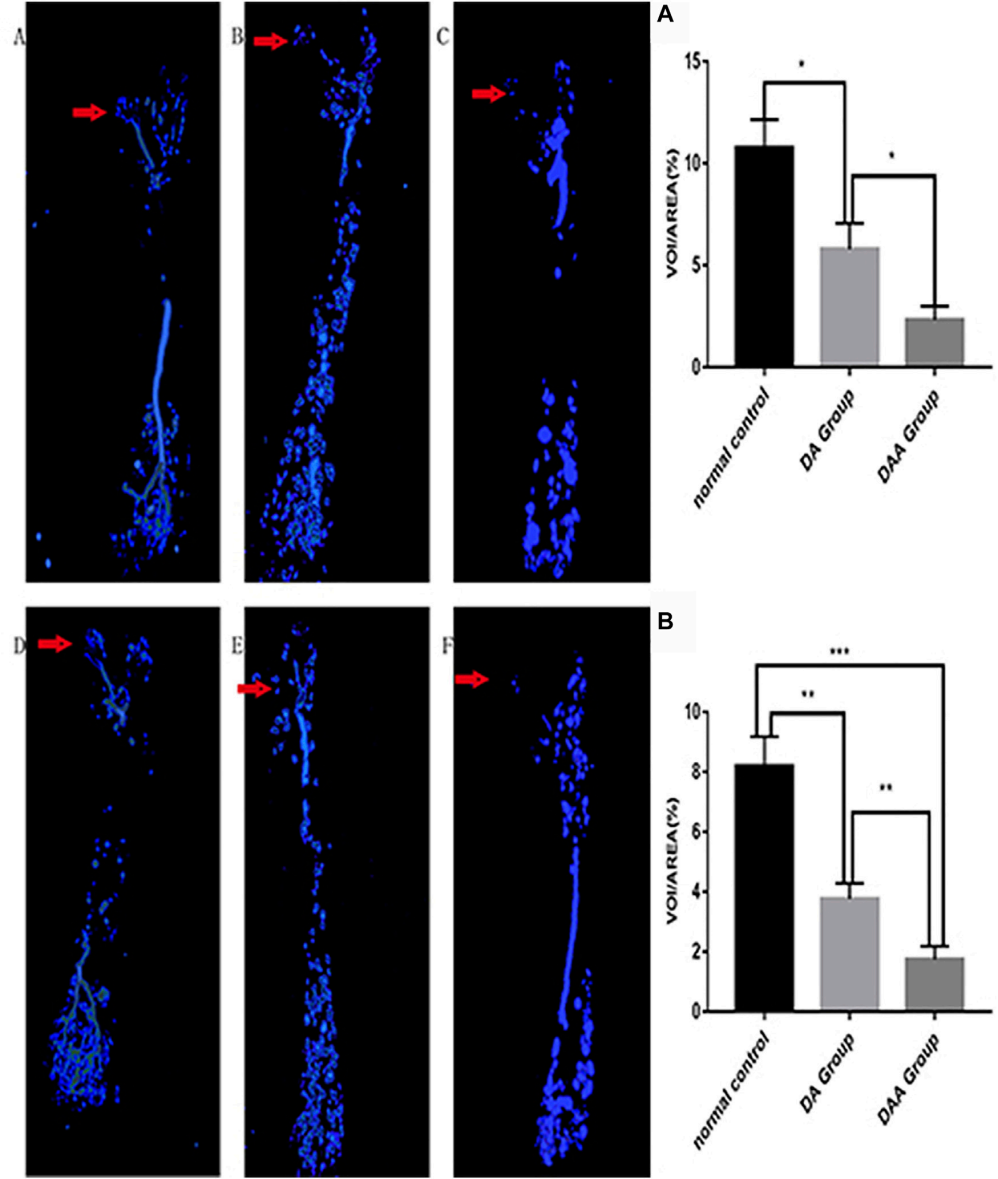
Analysis of blood vessels in the femoral head in the 4th **(A–C)** and 8th **(D–F) **weeks in the DAA group **(C,F)**, DA group **(B,E)**, and normal control group **(A,D)**. The red arrow indicates the area of the femoral head. Representative 3-D microangiographic images of blood vessels in the femoral heads of the mice in each group; the blue area represents the blood vessels in the femoral heads. Quantification of the total blood vessel volume in the femoral head [the number of femoral head vessels in three mice was statistically analyzed in the 4th **(A)** and 8th **(B)** weeks]. The data are presented as the mean ± standard deviation, *p < 0.05.

DAA reduced the number of local blood vessels in the femoral head of mice and increased the rate of osteonecrosis in the femoral head. As shown in Figure 3, in the 4th and 8th weeks, the DAA group had the smallest VOI/AREA value, and the normal control (NC) group had the largest VOI/AREA value. The blood vessels in the DAA group had a significantly lower volume than those in the DA group (*p* < 0.05). The NC group had higher blood vessel volumes than the DA group.

### Confirmation of the establishment of a femoral head osteonecrosis model based on the rates of osteonecrosis and mortality in mice

**FIGURE 4:**
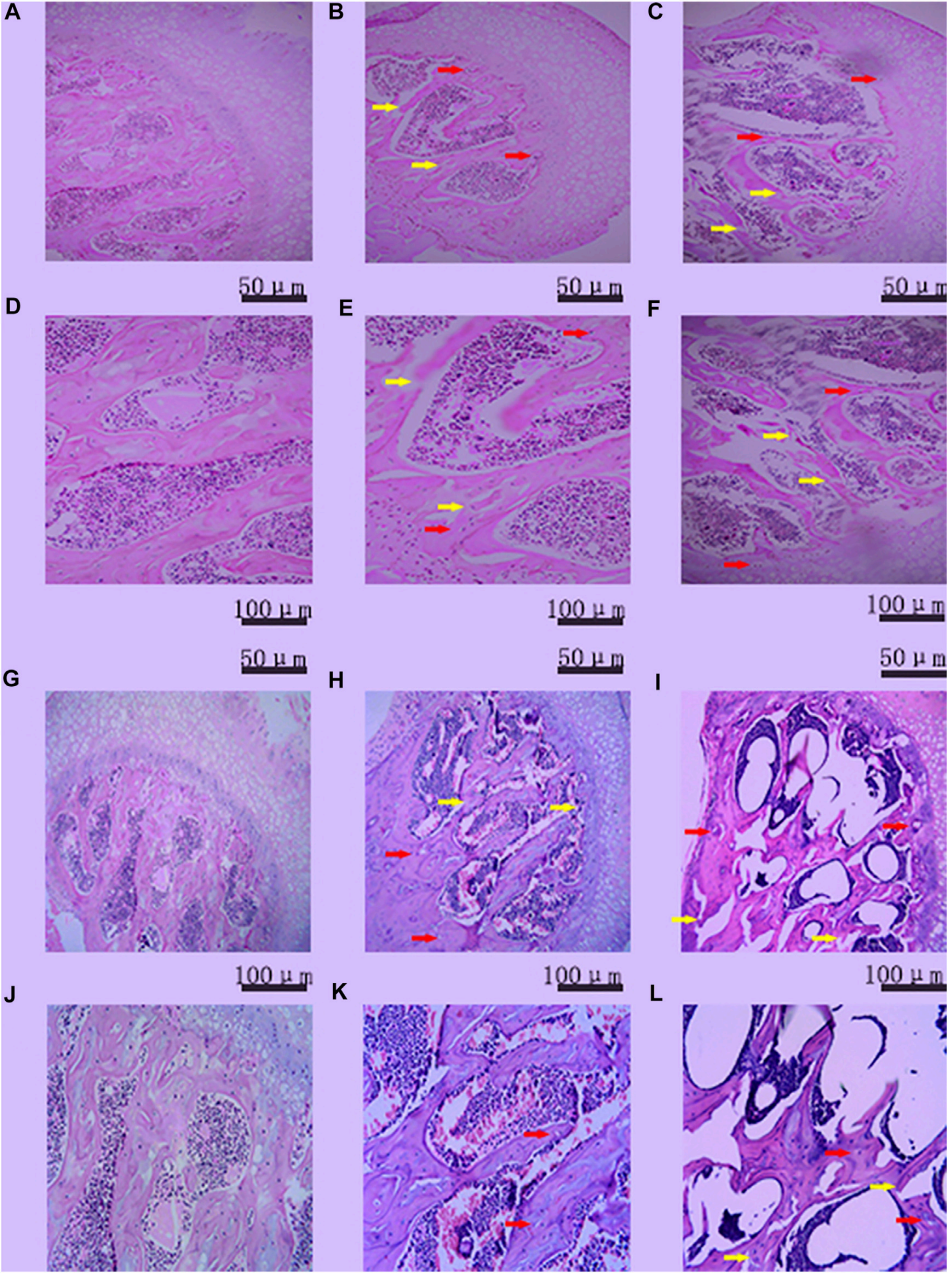
Histopathologicalanalysis of empty bone lacunae in the femoral head in the 4th **(A–F)** week and the 8th **(G–L)** week in the control group [**(A)** (100X), **(D)** (200X); **(G)** (100X), and **(J)** (200X)], DA group [**(B)** (100X), **(E)** (200X); **(H)** (100X), and **(K)** (200X)] and DAA group [**(C)** (100X), **(F)** (200X), **(I)** (100X), and **(L)** (200X)]. Empty bone lacunae (the red arrow) and trabecular fractures (the yellow arrow) were evident in the femoral heads of the three groups, demonstrating that the mouse model of femoral head osteonecrosis was successfully established.

DAA increased the rate of femoral head osteonecrosis in mice. As shown in Figure 4, the H&E staining results showed that more than three empty lacunae appeared in the femoral head region in the DA and DAA groups, and trabecular fractures were visible. These data demonstrate that the mouse model of femoral head osteonecrosis was successfully established (Theoleyre et al., 2004; Qin et al., 2006; Boyce and Xing, 2008; Okazaki et al., 2009; Singh et al., 2012; Dinh et al., 2017). There were no empty lacunae or trabecular fractures in the NC group, suggesting that there was no osteonecros is in the normal group. The success rate was 30% in the DA group and 50% in the DAA group at the 4th week. In the 8th week, no osteonecros is occurred in the NC group. The success rate was 40% in the DA group and 70% in the DAA group. The mortality rate of the mice in the DA group was 5% [0 (4th week) + 5% (8th week)], while that of the mice in the DAA group was 10% [0 (4th week) +10% (8th week)], and no mice died in the NC group. These data suggest that DAA can significantly increase the rate of femoral head osteonecrosis in mice, with no significant difference in mortality.

## Discussion

GCs are inexpensive and efficient, and they can quickly alleviate symptoms and diseases that are commonly seen in the clinic, such as asthma, systemic lupus erythematosus, rheumatoid arthritis, and allergic purpura. Despite their utility, GCs have been recognized as a major risk factor for femoral head osteonecrosis, with femoral head osteonecrosis occurring in up to 13% of patients on high doses (>3 g cumulative) of GCs (Assouline-Dayan et al., 2002; Lieberman et al., 2003; Chan et al., 2006; Mont et al., 2006; Law et al., 2008). To date, many international studies have been performed on the relationship between hormones (GCs) and femoral head osteonecrosis; however, an ideal animal model of femoral head osteonecrosis has not yet been established. Therefore, it is still necessary to establish a more efficient animal model of femoral head osteonecrosis (Xu et al., 2018). Mice are considered to be excellent experimental animals and are very suitable for the study of femoral head osteonecrosis, but there are few models of femoral head osteonecrosis in mice; the modeling methods include a high-dose GC model (Motomura et al., 2008; Dong et al., 2015; Kang et al., 2015; Yu et al., 2016), aGC combined with endotoxin model (Chen et al., 2014; Dong et al., 2015; Yu et al., 2016), aGC combined with allogeneic serum model (Tian et al., 2013; Wang et al., 2013), and an ASP combined with DEX model (Liu et al., 2016). These models all have shortcomings, such as low success rates and high death rates in the experimental animals.

GCs induce protein decomposition (Horber et al., 1985) and ultimately affect the weight of the mice. Weight is simply an indicator of health for mice. From the mouse body weight curves, we found that DAA affected body weight in the mice and significantly inhibited weight gain; however, the weight of the mice showed an overall increasing trend, which showed that the mice in each group were in good health condition. We thought the reason why did DAA significantly inhibit mouse weight gain might because the Ang II acts to enhance insulin sensitivity in a variety of animal models (Schinzari et al., 2018). No mice in the NC group died in this study, and the mortality rate of the mice in the DA group was 5% [0 (4th week) + 5% (8th week)], while that of the mice in the DAA group was 10% [0 (4th week) + 10% (8th week)]. The exact causes of death for these mice are unclear. He et al. (2016) applied DA to model femoral head osteonecrosis in mice. The death rate was 7.5%, and the success rate was 43% in that study; Yang et al. (2009) also applied DA to model femoral head osteonecrosis in mice. The success rate was 45% in that study; Wang et al. (2015) model femoral head osteonecrosis in mice by subcutaneously inject 21 mg/kg methylprednisolone for 4 weeks consecutively; Ryoo et al. (2014) model femoral head osteonecrosis in mice by chronic GC administration with co-treatment of LPS. Each approach has its own advantages and limitations.

Notably, DAA did not significantly influence mouse health or survival. Lipid metabolic disorder and hypercoagulation are considered two important causes of femoral head osteonecrosis. Studies by Fukushima et al. (2016) showed that increased secretion of various fat factors and an increased concentration of plasminogen activator inhibitor (pai-1) could significantly influence hemodynamic changes of the femoral head and the progression of bone osteonecrosis. Studies by Pritchett (2001) have shown that statins reduce the risk for osteonecrosis in steroid-treated patients. These findings indicate that lipid metabolism, high coagulation rates and femoral head osteonecrosis are closely related. With regard to the values of total cholesterol, total triglycerides and low-density lipoprotein in the 4th and 8th weeks in this study, the DAA group had the largest values, while the NC group had the smallest values. The difference in the values between the two groups was significant. This finding suggests that DAA can significantly affect lipid metabolism in mice and promote osteonecrosis. DAA also significantly influences clotting factors III, V, and Ⅹ (*p* < 0.05), but there is no obvious mechanism, and the causes are complex and unclear.

To date, there has been no report on vascular perfusion of the femoral head in mice; thus, this approach is a highlight of this experiment and will be helpful for subsequent studies. In this experiment, systemic perfusion imaging was performed on the mice through the heart. The results showed that the differences in blood transport in the femoral head between the mice in different groups were obvious, and the blood transport characteristics of bone osteonecrosis were clearly observed. It will be helpful to further study the characteristics of local blood transport in the femoral head; this approach provides an initial idea for judging osteonecrosis, although it is not currently an ideal method.

## Conclusion

In summary, our study is the first to present a method of modeling steroid-induced femoral head in mice using Ang II combined with ASP and DEX, and the success rate reached 70%. In this study on an Ang II intervention in the vascular system, ASP affected the blood system, which helped to simulate the environment of femoral head osteonecrosis in such diseases and better to serve clinical research on this type of disease. This experiment also has some deficiencies, one of which is few samples. Further optimization of this model is needed. However, it is expected that modeling of mouse femoral head osteonecrosis will be helpful for studying the pathogenesis of human femoral head osteonecrosis.

## Data Availability

The raw data supporting the conclusions of this article will be made available by the authors, without undue reservation.

## Abbreviations

Ang II, angiotensin II; ASP, asparaginase; DEX, dexamethasone; GC, glucocorticoid; DA, DEX + ASP; DAA, DEX + ASP + Ang II; NC, normal control; H&E, hematoxylin-eosin staining.
